# GLYX-13, a NMDA Receptor Glycine-Site Functional Partial Agonist, Attenuates Cerebral Ischemia Injury *In Vivo* and *Vitro* by Differential Modulations of NMDA Receptors Subunit Components at Different Post-Ischemia Stage in Mice

**DOI:** 10.3389/fnagi.2017.00186

**Published:** 2017-06-09

**Authors:** Chen Zheng, Zhi H. Qiao, Meng Z. Hou, Nan N. Liu, Bin Fu, Ran Ding, Yuan Y. Li, Liang P. Wei, Ai L. Liu, Hui Shen

**Affiliations:** Laboratory of Neurobiology in Medicine, School of Biomedical Engineering, Tianjin Medical UniversityTianjin, China

**Keywords:** transient middle cerebral artery occlusion, oxygen-glucose deprivation, ischemia, pathological synaptic plasticity, GLYX-13

## Abstract

Excessive activation of NMDA receptors (NMDARs) is implicated in pathological synaptic plasticity also known as post-ischemic long-term potentiation (i-LTP) which was produced by glutamate mediated excitotoxicity after stroke. In the past decades, many NMDARs inhibitors failed in clinical investigations due to severe psychotomimetic side effects. GLYX-13 is a NMDAR modulator with glycine site partial agonist properties and has potential protective effects on ischemic neuronal death. However, the underlying molecular mechanism of GLYX-13 attenuating the ischemic neuronal damage remains elusive. Our study was conducted to examine the molecular, cellular and behavioral actions of GLYX-13 in stroke, and further characterize the mechanism underlying the neuroprotective actions via modulation of the NMDAR subunit composition. In present study we found that *in vitro* oxygen-glucose deprivation (OGD) stroke model, GLYX-13 blocked i-LTP and restored the ratio of NR2A/NR2B subunit composition. The glycine site of NMDARs full coagonist D-serine completely blocked the effects of GLYX-13 on i-LTP. Besides, *in vivo* middle cerebral artery occlusion (MCAO) model, GLYX-13 decreased the cerebral infarct volume and reduced injury of hippocampus. Western analysis showed that GLYX-13 down-regulated the expression of phosphorylated NR2B (Tyr1472) and up-regulated phosphorylated NR2A (Tyr1325). Furthermore, GLYX-13 treatment along with NR2B specific antagonist (Ro256981) failed to exhibit any additional neuro-protective effects, whereas the application of NR2A antagonist (NVP-AAM007) abolished the neuroprotective effects of GLYX-13, which suggested that the protective action of GLYX-13 should be by its regulation of NMDAR subunit components. Our study provides important insights on the potential protective mechanism of GLYX-13 in ischemia and proposes the glycine site of NMDARs as a novel target for developing therapeutic strategies to store synaptic function in stroke.

## Introduction

Currently, focal cerebral ischemia (stroke) is one of the most common reasons of death and disability in middle-aged and elderly people worldwide (Lo, 2014). Compared to other tissues and organs in the body, the brain is particularly vulnerable to ischemic injury. A transient period (approximately 10 min) cerebral ischemia can produce profound neuronal damage immediately and continue progressively for months. Importantly, delayed and progressive nature of neuronal damage following focal cerebral ischemia points to a significant time window for stroke intervention and emphasizes the importance of ameliorating neuronal damage. Thus it would highlight new therapeutic targets for preventing the progression from ischemia to neuronal death (Lai et al., 2014).

Excitotoxicity is a major characteristic among the neurological diseases involving stroke (Vieira et al., 2016). One explanation for the peculiar susceptibility of brain to ischemia damage is that the brain tissue contains high levels of the excitatory neurotransmitter glutamate, and the neurons have amounts of corresponding receptors to respond to the neurotransmitters. One important relative hot perspective concerns the dual impacts of NMDA receptors (NMDARs) in neuronal survival and death (Lai et al., 2011). Recent evidence supports the notion that NR2A containing NMDARs in the neuronal synapse participate activity-dependent neuronal survival signaling, while NR2B containing NMDARs in extrasynaptic sites contribute to excitotoxicity following stroke (Martel et al., 2012). Therefore, many NMDAR antagonists were investigated in animal experiments and clinical trials in the past couple of years. However, these compounds have failed clinical tests in unexpected way mainly due to the major psychotomimetic side effects such as confusion, paranoia, depression and amnesia in subjects, and prevalence of neurological functions require these NMDARs as well.

GLYX-13 is a tetrapeptide derived from a monoclonal antibody which acts as a NMDAR partial agonist at the glycine site with therapeutic potential to improve learning and cognition (Moskal et al., 2005). Besides, GLYX-13 is permeable to blood brain barrier and has been proved to increase activity-dependent synaptic plasticity (Schaffer-collateral CA1 LTP *in vitro*; Moskal et al., 2005). In addition, a preliminary study reported that GLYX-13 has neuroprotective potential on Mongolian gerbils suffering from bilateral carotid occlusion and reduced delayed (24 h) injury of CA1 pyramidal neurons (Stanton et al., 2009). In consideration of the modulation effect on NMDARs by GLYX-13 and the alterations of NMDAR subunits expression following transient ischemia (Liu et al., 2010), it is possible that GLYX-13 exerts neuroprotective effects on stroke through modulation of the NMDARs.

In this study we investigated the impact of GLYX-13 on both middle cerebral artery occlusion (MCAO) and oxygen-glucose deprivation (OGD) induced neuronal injury *in vivo* and *in vitro* respectively, and we explore the possible neuroprotective mechanism of GLYX-13 on ischemic injury, as a glycine site partial agonist of NMDARs through the modulation of NMDARs subunit components.

## Materials and Methods

### Animals and Housing

Two months old male C57BL/6 mice (20–25 g) were used in the study. Animals were purchased from the institute of zoology, Chinese academy of sciences. Animals were housed in cages with wood shaving bedding and were maintained on a 12:12 light:dark cycle in the Tianjin Medical university of China-approved animal facility. Animals were given enough food and water throughout the studies unless otherwise noted. All experiments were approved by the Animal Care and Use Committee of Tianjin medical university, in compliance with National Institutes of Health guidelines.

### *In Vivo* Middle Cerebral Artery Occlusion Ischemia Model

Transient MCAO was adopted in our study, mainly according to the method of Rousselet et al. (2012). Briefly, 20–25 g male mice were anesthetized with isoflurane. During the whole surgery body temperature of the mice is maintained constant by a heating pad (Rousselet et al., 2012). A monofilament suture (about 9–10 mm is coated silicon, Doccol Corporation) was used to occlude the right MCA. Heart rate, rectal temperature, and blood gases were monitored during the MCAO surgery. All the mice were subjected a 1 h MCAO. For sham group, all procedures of operation were same except that the MCAO suture was not inserted into MCA. For drug treatment experiments, drugs were then performed via intraperitoneal injection 2 h after the onset of MCAO (GLYX-13, Tocris, 10 mg/kg, D-serine, Sigma, 50 mg/kg, Ro256981 Tocris 5 mg/kg, NVP-AAM077 Tocris 2.4 mg/kg).

### Measurement of Cerebral Blood Flow

Cerebral blood flow was measured by Laser Doppler Flowmetry (Perimed, PeriFlux5010, Sweden) with a straight laser Doppler probe (Perimed, Probe 418, Sweden) as previously described (Iwai et al., 2004). The probe was placed in the territory supplied by the MCA (AP-1.0 mm, L5.0 mm from bregma). Relative cerebral blood flow was measured before MCAO (baseline) and during occlusion. Changes of cerebral blood flow were expressed as percentage of the baseline value. Only those mice which ipsilateral regional cerebral blood flow ≤20% of baseline were used for further study.

### Neurological Assessment

Twenty-four hours after reperfusion, neurological score was assessed. Neurological score contributed to evaluate the success of transient MCAO and to estimate the degree of neurological deficits and injury severity. Neurological assessments were scored as previously described (Jiang et al., 2005).

0: Normal.
1: Mild circling behavior, left forepaw cannot be fully extended.
2: Mild consistent circling, and walking to the paralyzed side.
3: Strong circling and holds a rotation position more than 2 s with its nose almost reaching its tail.
4: Strong circling progresses into severe rotation, can not walk spontaneously and miss the contralateral reflex activity.
5: Coma or dying.

### Calculation of Infarct Volumes

Twenty-four hours after reperfusion, the animals were decapitated and the brains were removed into a mouse brain matrix for 1 mm section. Then sections were immersed in 1% triphenyltetrazolium chloride (TTC, Sigma) in PBS and incubated about 15 min at 37°C. The area of unstained brain was identified as the infarct area. The infarct area was measured using the image pro plus software 6.0. The infarct volume was reckoned by multiplying summed infarct area from each section by the slice thickness. Take the edema after ischemia into consideration, the infarct volume were expressed as percentage of hemisphere volume (Yang et al., 1998).

### Brain Tissue Preparation and Staining

The animals were sacrificed 24 h after reperfusion. The brain was fixed using 4% paraformaldehyde and then was embedded in paraffin, and was cut into coronal sections of 5 μm thickness for histological evaluation.

#### Hematoxylin and Eosin (HE) Staining

Paraffin embedded brain sections were de-waxed and rehydrated, and stained with eosin (HE). The histological staining results were measured under light microscopy and used to locate the hippocampal regions for observation.

#### Fluoro Jade C Staining

To visualize degenerating neurons, Fluoro Jade C (FJC) staining was performed according to the instruction of manufacturer (Millipore AG325). After a series of deparaffinating, rehydration, brain sections were incubated in 0.06% potassium permanganate for 10 min, rinsed in distilled water for 2 min, and then were stained prior to 0.0004% FJC in 0.1% acetic acid and protected from light and counted (Balsara et al., 2015).

### Western Blot

Western blot was done as previously described (Zhao et al., 2013). Briefly, equal amounts of prepared sample protein (ipsilateral hippocampus *n* = 6) were separated by 10% SDS-PAGE and then were transferred to polyvinylidene fluoride (PVDF) membranes. The membranes were blocked by 5% BSA or skimmed milk for 1 h, then were incubated with primary antibodies (mouse anti-NMDAR2A 1:500, mouse anti-NMDAR2B 1:500 BD Transduction Laboratories; rabbit anti-NMDAR2A phospho Y1325 1:1000, rabbit Phospho-NMDAR2B Tyr1472 1:1000 Cell Signaling; β-actin 1:2000 Santa Cruz) at 4°C overnight. β-actin was used as a loading control. Next the membranes were incubated with anti-rabbit, anti-mouse secondary antibodies. To visualize the reaction, the enhanced chemiluminescence (ECL) detection reagents (Beyotime Biotechnology) were used. The intensity of blots was quantified with image J software.

### Hippocampal Slice Preparation

The preparation of brain slices were performed as previously described (Shen et al., 2007; Yao et al., 2012). Briefly, 4 week old male C57BL/6 mice were sacrificed rapidly. The whole brain were removed and 320 μm thickness coronal slices were cut by a vibratome in ice, ACSF bubbled continuously with 95% O_2_/5% CO_2._ The brain slices were incubated in a chamber with above ACSF at 32°C for 1 h recovery.

### Electrophysiological Studies

Whole cell patch clamp recording were performed. The pipettes solution containing (in mmol/L): 130 CsMeSO_3_, 8 NaCl, 10 HEPES, 5 QX314, 4 Mg-ATP, 0.3 Na-GTP, 0.2 EGTA, meanwhile PH was adjusted to 7.2 by CsOH and osmolarity was maintained to 275–290 mOsm (Massey et al., 2004). The pyramidal neurons of CA1 region were visualized with an upright microscope (BX51-WI, Olympus, Japan) and an infrared video camera (710 M, DVC, Pleasant Hill, CA, USA). Recording were conducted with a 2 kHz Bessel filter at a 10 kHz sampling frequency using Axon patch 200B patch-clamp amplifier (Molecular Devices, Foster City, CA, USA) and Digidata 1440 interface (Molecular Devices, Foster City, CA, USA). Series resistance was monitored during the experiments and cells that showed >20% change were removed from subsequent analysis. If the series resistance was greater than 30 MΩ, recordings were also discarded from analysis (Dennis et al., 2011). Synaptic responses were induced at 0.05 Hz by a tungsten bipolar electrode (FHC, USA) which was placed in the stratum radiatum (for Schaffer collateral stimulation); meanwhile the recording pipette was located in the pyramidal cell layer of CA1 (Dennis et al., 2011). Evoked Excitatory postsynaptic current (eEPSC) were recorded at −70 mV holding potential in ACSF perfusion containing bicuculline methiodide (10 μmol/L, sigma) to block GABA_A_ receptor-mediated inhibitory synaptic currents (Yao et al., 2012). NMDAR mediated EPSCs were recorded in Mg^2+^-free ACSF and clamped at +40 mV in the presence of NBQX (5 μmol/L) and bicuculline methiodide (10 μmol/L) to block AMPAR and GABA_A_R respectively.

OGD was produced by perfusing slices with ACSF saturated with 95% N_2_/5% CO_2_ and replacing the 10 mmol/L glucose with 10 mmol/L sucrose (Dennis et al., 2011).

### Statistical Analysis

Results were presented as mean ± SEM. Mann-Whitney *U*-test was used in analysis of Neurological scores. One-way analysis of variance (ANOVA) with a *post hoc* analysis (Newman-Keuls) for multiple comparisons was applied to determine the statistical differences between three or more groups. Differences were considered significant when *p* < 0.05.

## Results

### Physiology

There were no significant difference in heart rate, the pH of blood, PO_2_, PCO_2_ between vehicle and GLYX-13 treated group during MCAO surgery (Table 1).

**Table 1:** Physiological variables in control and GLYX-13 treated group.

Physiological variable	Vehicle	GLYX-13
Heart rate, bpm	451 ± 9	453 ± 6
Rectal temperature	37.3 ± 0.15	37.4 ± 0.11
pH	7.22 ± 0.01	7.21 ± 0.02
PO_2_, mmHg	108.1 ± 9	107.2 ± 11
PCO_2_, mmHg	54.6 ± 3	53.8 ± 5

### GLYX-13 Treatment Reduced Neurological Severity Scores in MCAO Mice

**Figure 1:**
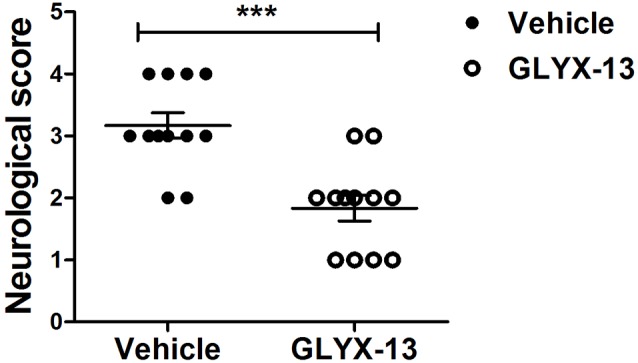
Effect of GLYX-13 on Neurological function. Neurological score were evaluated 24 h after reperfusion. Statistical analysis was determined by Mann-Whitney *U*-test, *n* = 12, ***p < 0.001.

We evaluated neurological score 24 h after reperfusion. The neurological function were significantly improved in GLYX-13 treatment group in comparison with the vehicle group (Figure 1; GLYX-13 1.8 ± 0.2 vs. Vehicle 3.2 ± 0.2, *n* = 12, ***p < 0.001).

### GLYX-13 Decreased the Infarct Volume in MCAO Mice

**Figure 2:**
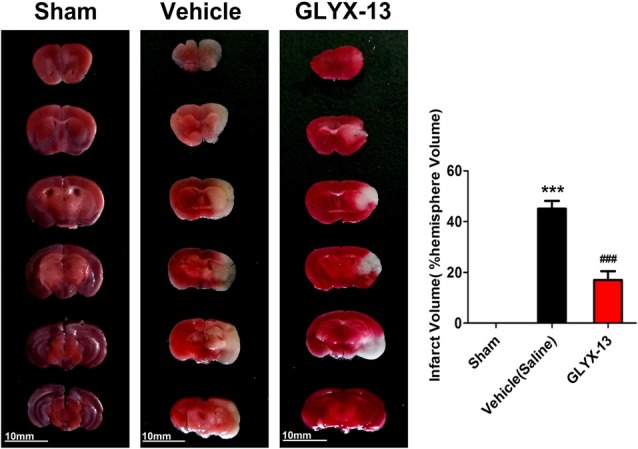
GLYX-13 treatment reduced ischemic infarct volume *in vivo* middle cerebral artery occlusion (MCAO) model. Cerebral infarct volume was assessed via triphenyltetrazolium chloride (TTC) staining of coronal sections 24 h after reperfusion in representative mice. The infarct volume was analyzed in each group. Data were expressed as mean ± SEM (*n* = 12 animals per group). One-way analysis of variance (ANOVA) and Newman-Keuls post test, ***p < 0.001, vs. Sham; ^###^*p* < 0.001, vs. vehicle.

We explore the possible neuroprotective effects of GLYX-13 *in vivo* MCAO model. The cerebral infarct volume was evaluated by TTC staining 24 h after reperfusion. Normal brain tissue was stained red, while the infarction remained unstained (white). Compared with the vehicle group, the infarct volume exhibited remarkable decrease in the GLYX-13 group (Figure 2; GLYX-13 17 ± 3.4% vs. Vehicle 45.0 ± 3.1%, ^###^*p* < 0.001).

### GLYX-13 Treatment Reduced Injury of Neurons in Hippocampus Caused by MCAO

**Figure 3:**
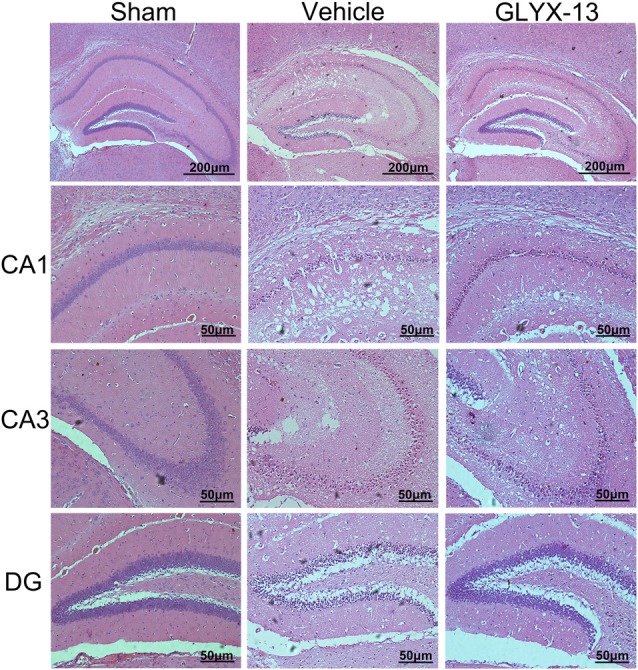
Administration of GLYX-13 attenuating post-ischemic neuronal injury. Representative hematoxylin and eosin (HE) staining coronal brain sections showed the hippocampal CA1, CA3, dentate gyrus (DG) areas, respectively.

**Figure 4:**
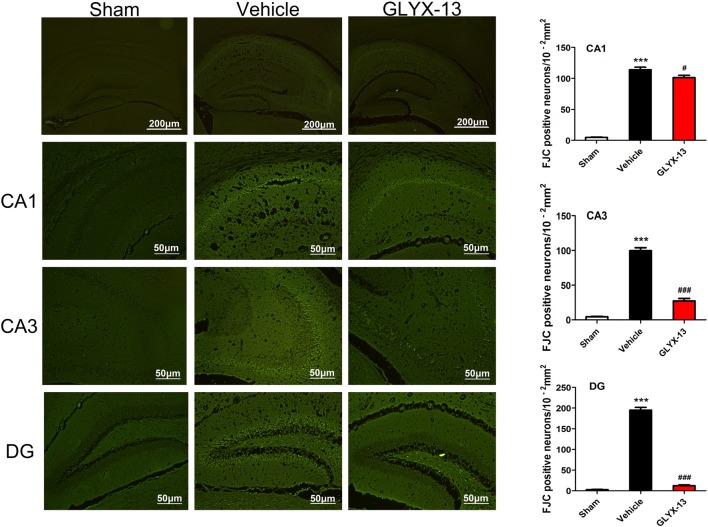
Administration of GLYX-13 dramatically decreased the number of Fluoro Jade C (FJC) positive neurons in hippocampus. Three regions (CA1, CA3 and DG) were further evaluated to compare the FJC fluorescent signals between sham, vehicle and GLYX-13 treated group. Photomicrographs of FJC-stained three regions of interest were shown in each group. GLYX-13 treatment dramatically reduced the number of FJC positive neurons in DG and CA3 region (^###^*p* < 0.001) and also reduced the number of FJC positive neurons in CA1 (^#^*p* < 0.05). The numbers of FJC positive neurons were expressed as the mean number per field of view. Data were expressed as mean ± SEM (*n* = 5); one-way ANOVA and Newman-Keuls post test, ***p < 0.001, vs. Sham; ^###^*p* < 0.001, ^#^*p* < 0.05, vs. vehicle.

HE staining was performed for histological evaluation within hippocampus (Figure 3). FJC staining was also performed to further assess the neuronal injury. FJC positive neurons were with strong green fluorescent signal. Notably, there was a dramatic decrease in the number of FJC positive neurons in GLYX-13 treated group in dentate gyrus (DG; GLYX-13 12.4 ± 4.2/10^−2^mm^2^ vs. Vehicle 195 ± 14.2/10^−2^mm^2^, *n* = 5, ^###^*p* < 0.001) and CA3 region (GLYX-13 27.2 ± 7.6/10^−2^mm^2^ vs. Vehicle 99.4 ± 9.7/10^−2^ mm^2^, *n* = 5, ^###^*p* < 0.001), moreover, there was still a decrease in CA1 region in GLYX-13 treated group (GLYX-13 101.3 ± 7.4/10^−2^ mm^2^ vs. Vehicle 118.3 ± 8.3/10^−2^ mm^2^, *n* = 5, ^#^*p* < 0.05; Figure 4).

### GLYX-13 Down-Regulated the Phosphorylated Level of NR2B and Up-Regulated the Phosphorylation of NR2A

**Figure 5:**
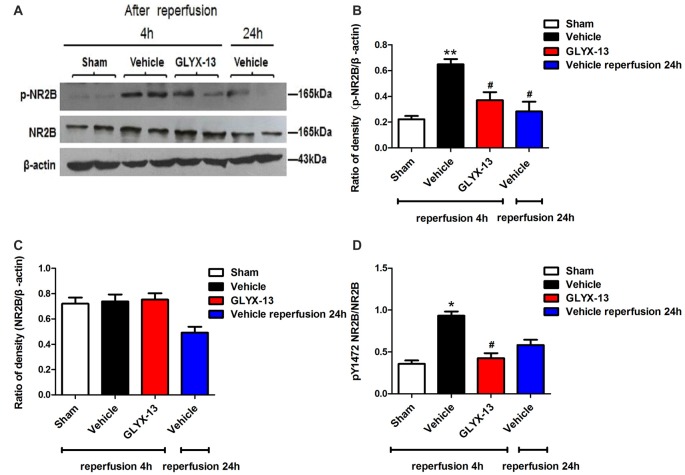
GLYX-13 reduced the expression of pY1472 NR2B 4 h after reperfusion. **(A)** Representative images of western blot. The intensity of each band was quantified by image j and normalized in relation to β-actin. **(B–D)** Statistical analysis of densitometric data from indicated experimental groups. The values are expressed as mean ± SEM, *n* = 3 independent experiments. One-way ANOVA and Newman-Keuls post test, *p < 0.05, **p < 0.01, vs. Sham; ^#^*p* < 0.05, vs. Vehicle.

**Figure 6:**
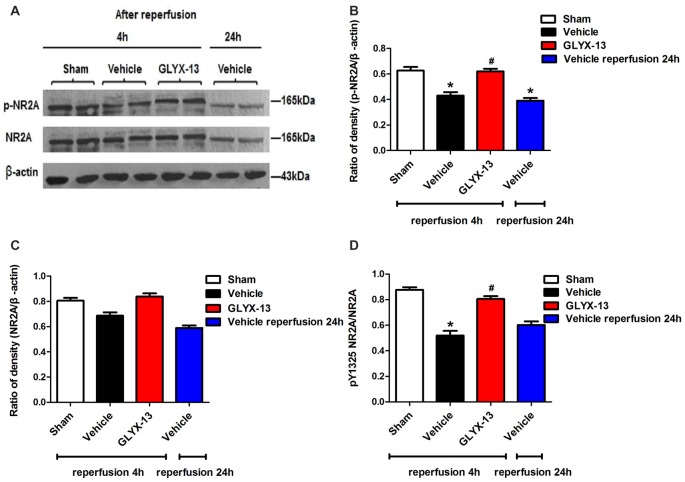
GLYX-13 increased the expression of pY1325 NR2A 4 h after reperfusion. **(A)** Representative images of western blot. The intensity of each band was quantified by image j and normalized in relation to β-actin. **(B–D)** Statistical analysis of densitometric data from indicated experimental groups. The values are expressed as mean ± SEM, *n* = 3 independent experiments. One-way ANOVA and Newman-Keuls post test, *p < 0.05, vs. Sham; ^#^*p* < 0.05, vs. Vehicle.

**Figure 7:**
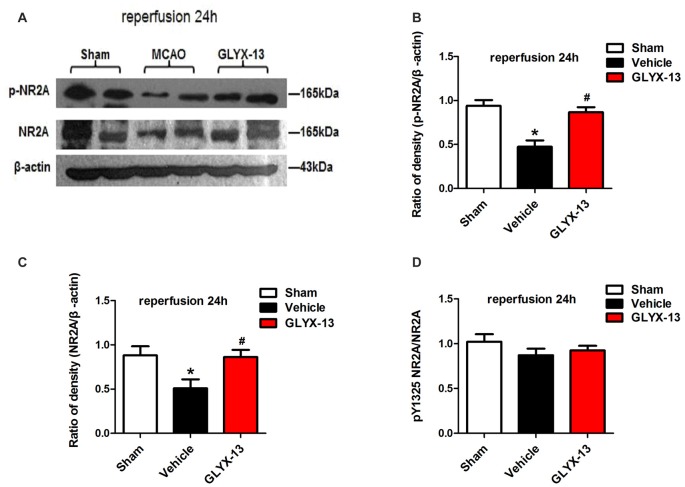
GLYX-13 ameliorated the loss of NR2A after 24 h reperfusion. **(A)** Representative images of western blot. The intensity of each band was quantified by Image j and normalized in relation to β-actin. **(B–D)** Statistical analysis of densitometric data from indicated experimental groups. The values are expressed as mean ± SEM, *n* = 3 independent experiments. One-way ANOVA and Newman-Keuls post test, *p < 0.05, vs. Sham; ^#^*p* < 0.05, vs. Vehicle.

**Figure 8:**
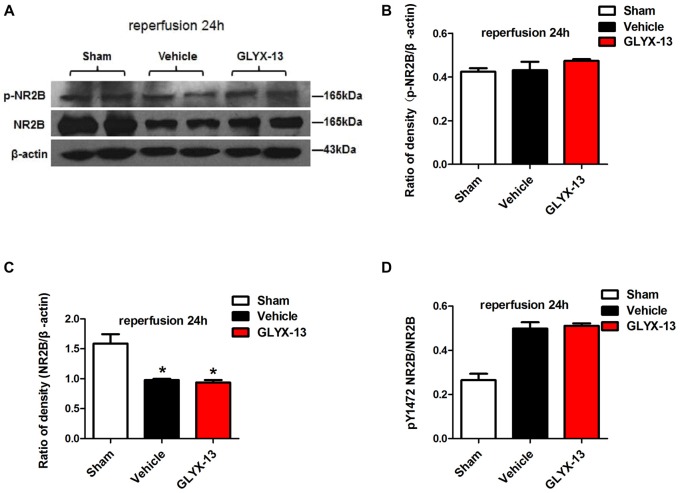
GLYX-13 has no effect on the loss of NR2B after 24 h reperfusion. **(A)** Representative images of western blot. The intensity of each band was quantified by image j and normalized in relation to β-actin. **(B–D)** Statistical analysis of densitometric data from indicated experimental groups. The values are expressed as mean ± SEM, *n* = 3 independent experiments. One-way ANOVA and Newman-Keuls post test *p < 0.05, vs. Sham.

NR2A and NR2B containing NMDARs are linked to different intracellular cascades and play the opposite roles in neuronal survival and death during ischemia, which is the core composition of subtype hypothesis of NMDARs. Thus activation of the synaptically localized NR2A-containing NMDARs promotes neuronal survival and exerts a neuroprotective potential. In contrast, activation of extra-synaptic NR2B containing NMDARs mainly result in excitotoxicity (Lai et al., 2011). It was reported that phosphorylated NR2A (Tyr1325) and phosphorylated NR2B (Tyr1472) were most relevant site to synaptic activity and excitotoxicity (Wu et al., 2013). Therefore, we focused on the expression of NR2A and NR2B and their phosphorylation at site Tyr1325 and Tyr1472 respectively after ischemia. Western blot analysis showed the level of p-NR2B (Tyr1472) in hippocampal tissue exhibited a marked increase after reperfusion 4 h (Vehicle 0.6488 ± 0.04057 vs. Sham 0.2221 ± 0.02572, **p < 0.01, *n* = 3 independent experiments), whereas the expression of p-NR2A (Tyr1325) reduced compared with sham group (Vehicle 0.4299 ± 0.02689 vs. Sham 0.6264 ± 0.02831, *p < 0.05, *n* = 3 independent experiments). No significant difference was found in NR2A and NR2B between vehicle and sham after reperfusion 4 h. Notably, in this study we found that GLYX-13 alleviated excitotoxicity through decreasing the tyrosine phosphorylation of NR2B subunit at site Tyr1472 after reperfusion 4 h (GLYX-13 0.3708 ± 0.06223 vs. vehicle 0.6488 ± 0.04057, ^#^*p* < 0.05, *n* = 3 independent experiments), which site was reported closely related to neuronal death before Li et al. (2017). Meanwhile, GLYX-13 treated group had a greatly higher level of p-NR2A expression than vehicle (GLYX-13 0.6190 ± 0.02101 vs. vehicle 0.4299 ± 0.02689, ^#^*p* < 0.05). Moreover the ratio of pY1472 NR2B/total NR2B and pY1325 NR2A/total NR2A were also restored by GLYX-13 treatment (Figures 5, 6). Furthermore, GLYX-13 ameliorated the loss of NR2A (GLYX-13 0.8613 ± 0.08200 vs. vehicle 0.5096 ± 0.1002, ^#^*p* < 0.05, *n* = 3 independent experiments) 24 h after reperfusion (Figure 7). However, GLYX-13 had no significant effect on the expression of NR2B 24 h after reperfusion (Figure 8).

### Activation of NMDAR Coagonist Site and the Neuroprotective Effects on Pathological Plasticity by GLYX-13

**Figure 9:**
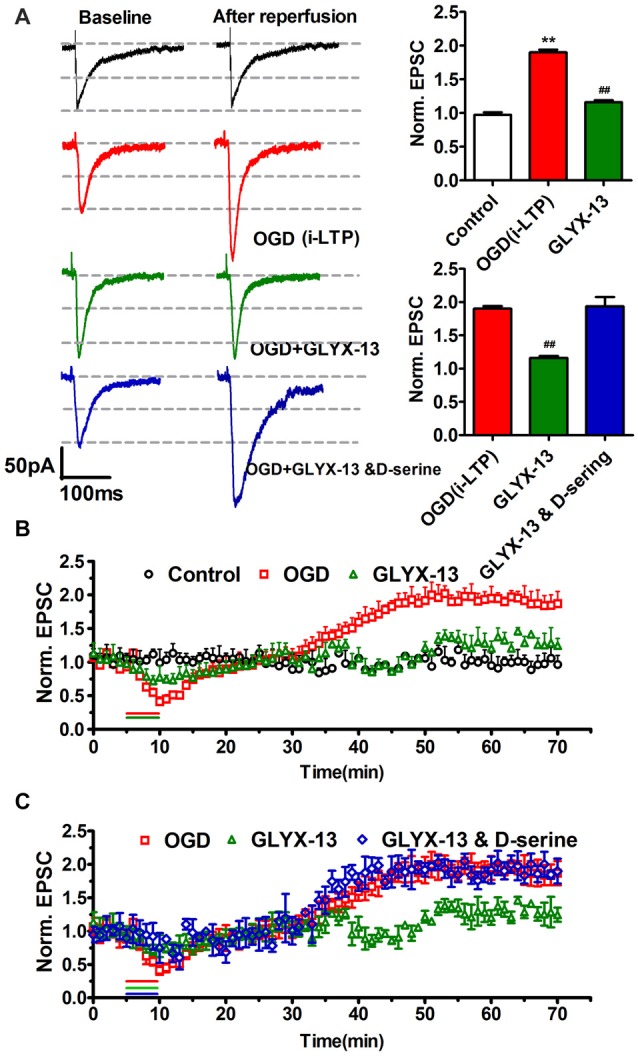
GLYX-13 ameliorated pathological potentiation of excitatory postsynaptic current (EPSC). **(A)** Sample traces were presented; bar chart of the data showed the effects of GLYX-13 on ischemic long-term potentiation (i-LTP; *n* = 5, one-way ANOVA and Newman-Keuls post test, **p < 0.01, compared with control; ^##^*p* < 0.01, compared with oxygen-glucose deprivation (OGD) group). **(B)** The changes in pathological i-LTP were under various conditions. GLYX-13 at 10 μmol/L completely abolished i-LTP. As a control, no obvious change in EPSCs was observed when hippocampal slices were not suffered from OGD treatment. **(C)** Suppression of i-LTP induced by GLYX-13 was reversed by full glycine site agonist D-Serine (100 μmol/L).

We adopted whole-cell patch clamp to record eEPSC in CA1 neurons of hippocampal slice when cells were clamped at −70 mV. OGD induced a significant increase of eEPSC (OGD 190.1 ± 3.9% of control, *n* = 5, **p < 0.01, one-way ANOVA; Figure 9). However this pathological potentiation could be abolished by selective NMDAR antagonist AP5, which implied that i-LTP required the participation of NMDARs (Figure 11). Notably, the application of 10 μmol/L GLYX-13 ameliorated pathological potentiation of eEPSC (GLYX-13 116.1 ± 3.0% vs. OGD 190.1 ± 3.9%, *n* = 5, ^##^*p* < 0.01, Figure 9). To detect whether the inhibition of i-LTP by GLYX-13 was because of binding to the glycine co-agonist site on the NMDARs, 100 μmol/L full glycine site agonist D-Serine was used, which concentration was confirmed to fully stimulate the glycine site (Zhang et al., 2008). The application of 100 μmol/L D-serine completely occluded the effect of GLYX-13 (D-serine 193.7 ± 3.1% vs. GLYX-13 116.1 ± 3.0%, *n* = 5, *p* < 0.01, Figure 9). The above results suggested that GLYX-13 exerts inhibitory effect on pathological synaptic plasticity by binding the glycine co-agonist site of NMDARs.

**Figure 10:**
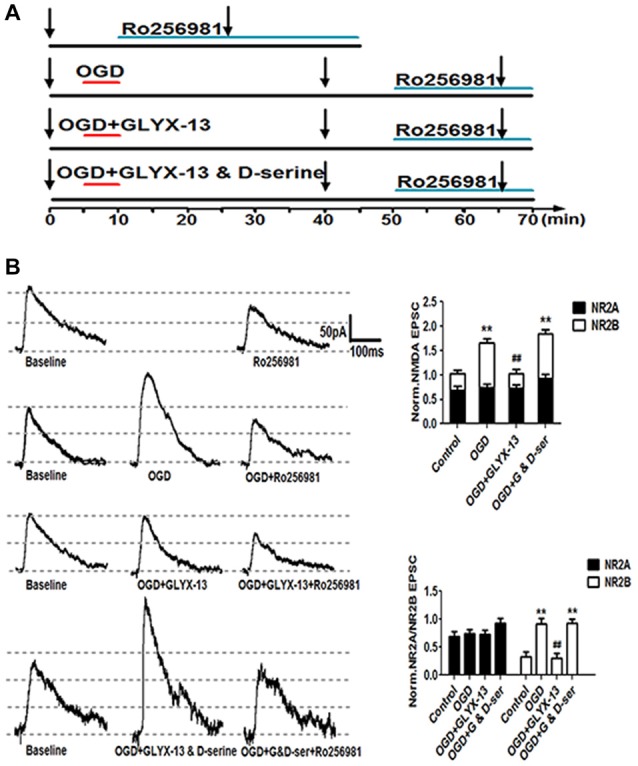
GLYX-13 suppressed the elevation of NR2B-containing NMDA receptors (NMDARs) component induced by OGD treatment. **(A)** Schematic paradigms showed experimental protocols adopted to detect the NR2 subunit components under control, OGD, OGD with 10 μmol/L GLYX-13, OGD with co-application of GLYX-13 and D-serine (100 μmol/L). Selective NR2B subunit antagonist Ro256981 (20 nmol/L) was used to exhibit the amplitude NR2A and NR2B (total amplitude subtract NR2A) containing NMDARs mediated EPSCs in CA1 neurons. The arrows in the paradigm represent the time points to record EPSCs. **(B)** Sample traces were presented and normalized NMDA EPSCs of total NMDA EPSCs, NR2A and NR2B-containing NMDAR subunit component under various conditions. (*n* = 5, one-way ANOVA and Newman-Keuls post test, **p < 0.01, compared with control; ^##^*p* < 0.01, compared with OGD group).

**Figure 11:**
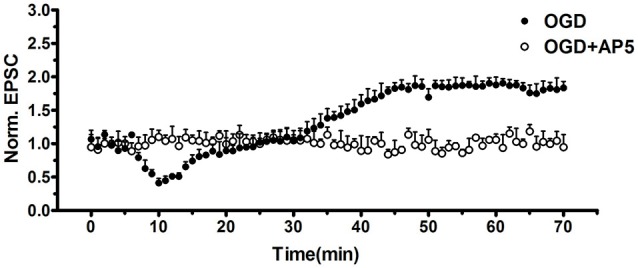
OGD-induced i-LTP was completely abolished by selective NMDAR antagonist AP-5 (50 μmol/L, *n* = 5, student’s *t*-test, *p* < 0.01).

### GLYX-13 Regulated the NMDAR Subunit Components which Have Been Already Changed by OGD

Considering the results of western blot analysis, we have found that GLYX-13 could influence the expression of NR2A and NR2B and the phosphorylation of above subunits. Accordingly, we hypothesized that GLYX-13 probably inhibit the pathological potentiation of eEPSC by modulating the subunit composition of NMDARs. Therefore, we carried out a series of experimental protocols and use full of the selective NR2B antagonist Ro256981 (20 nmol/L) to investigate the functional NR2A and NR2B-containing NMDAR mediated eEPSC (NR2A/NR2B-eEPSC) amplitude under various conditions (Figure 10A). We observed that OGD induced a significant increase of the amplitude of NR2B-eEPSC (OGD 277.4 ± 9.1% of control) with the enhancement of total NMDAR-eEPSC (OGD 162.8 ± 8.0% of control, *n* = 5, **p < 0.01; Figure 10B). However, we only found a slight but not significant increase in NR2A-eEPSC. Notably, simultaneous giving 10 μmol/L GLYX-13 during OGD effectively suppressed the pathological potentiation of NR2B-eEPSC (GLYX-13 91.3 ± 8.3% vs. OGD 162.8 ± 8.0%, *n* = 5, ^##^*p* < 0.01). Moreover, 100 μmol/L full glycine site co-agonist D-serine entirely reversed the inhibiting effect on NR2B-eEPSC produced by GLYX-13 (D-serine 278.3 ± 7.9% vs. GLYX-13 91.3 ± 8.3%, *n* = 5, **p < 0.01; Figure 10B). Consequently, GLYX-13 restored the NR2B subunit-mediated EPSC back to normal level.

### GLYX-13 Play Neuroprotective Role *In Vivo* MCAO Mice Also via Increasing the NR2A Components

**Figure 12:**
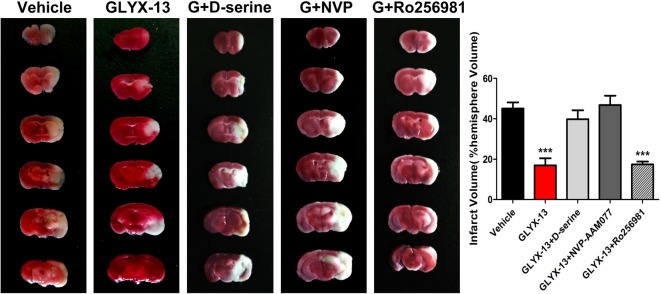
GLYX-13 reduced cerebral infarct volume through enhancing NR2A-containing components and decreasing NR2B-containing components *in vivo* MCAO ischemia model. Representative brain sections were stained with TTC under various treatments. Unstained area is infarct. GLYX-13 treatment along with selective NR2B antagonist (Ro256981 5 mg/kg) 2 h after 1 h MCAO challenge failed to display any additional neuroprotective effects; by contrast GLYX-13 treatment along with selective NR2A antagonist (NVP-AAM077 2.4 mg/kg) reversed the amelioration of infarct volume produced by GLYX-13 (*n* = 6, ***p < 0.001, compared with Vehicle).

Above results of electrophysiological study suggested that GLYX-13 exerts neuro-protective effects *in vitro* OGD model, to a great extent which mimics the acute situation after stroke attack. However, it is necessary to verify whether modulation of NMDAR NR2 subunits would also be effective to alleviate cerebral ischemic injury *in vivo*. TTC staining showed that treatment of GLYX-13 + Ro-2569819 (selective NR2B antagonist 5 mg/kg) after MCAO failed to present additional neuro-protective effect compared to GLYX-13 intervention alone (G + R 17.45 ± 1.442% vs. GLYX-13 17 ± 3.468% *n* = 6, *p* > 0.05); by contrast treatment of GLYX-13 + NVP-AAM077 (selective NR2A antagonist 2.4 mg/kg) completely reversed the amelioration of cerebral infarction elicited by GLYX-13 (G + N 46.79 ± 4.620% vs. GLYX-13 17 ± 3.468%; Figure 12). Above results suggested that compared with the principal role of NR2B subunits *in vitro* acute OGD model, the dysfunction of NR2A subunits are likely more obvious *in vivo* a few hours even a few days after stroke.

## Discussion

Stroke is a common reason of death and disability all over the world, and has become a heavy financial burden to families and society (Gladstone et al., 2002). Since neuronal death in the brain following stroke is a prolonged process, exploring the underlying injury mechanism helps discovering the novel therapies that alleviate neuronal damage even when administered several hours to days after attack (Lai et al., 2014).

Numerous studies have reported the apparent effects of NMDAR modulator GLYX-13 on antidepressant treatment (Burgdorf et al., 2013, 2015; Preskorn et al., 2015; Cooper et al., 2016; Lepack et al., 2016; Yang et al., 2016; Liu et al., 2017). However, there have been only a few researches focusing on the neuroprotective effects of GLYX-13 on ischemia injury or stroke. Stanton et al. (2009) reported that GLYX-13 potently reduced delayed neuronal death of CA1 pyramidal neurons of Mongolian gerbils produced by bilateral carotid occlusion model. Besides Zhang et al. (2015) discovered that GLYX-13 protected synaptic connections in the brain from structural change induced by cortical spreading depolarization which was generally accepted associated with stroke and traumatic brain injury.

In the present study, we found that glycine-binding site partial agonist GLYX-13 alleviated ischemic injury through down-regulating the NR2B subunit composition and up-regulating that of NR2A *in vivo* MCAO Model. Besides, GLYX-13 ameliorated pathological plasticity (post-ischemic LTP) and exerted neuroprotective effect via resetting the NR2B-containing NMDAR to control levels *in vitro* OGD induced ischemia model. Moreover, the application of full coagonist D-serine completely blocked the effects of GLYX-13 on i-LTP, indicating that GLYX-13 acted by modulating the glycine site of NMDARs. Early studies have shown that selective inhibition of NR2B-containing NMDARs were highly neuro-protective in models of cerebral ischemia and other neurodegenerative diseases whereas selective inhibition of the NR2A-containing NMDARs exacerbated neuronal death (Kim et al., 2005; Liu et al., 2007; Chen et al., 2008). In view of the above study evidences, the remodulation of NMDAR subunit components by GLYX-13 was a reasonable explanation for the protective action of GLYX-13. Furthermore, GLYX-13 treatment along with selective NR2B antagonist (Ro256981) failed to display any additional neuroprotective effects; by contrast GLYX-13 treatment along with selective NR2A antagonist (NVP-AAM077) reversed the amelioration of infarct volume produced by GLYX-13 alone. All of the above experimental evidences further verified that GLYX-13 exerts neuro-protective effects on cerebral ischemic injury mainly via the remodulation of NMDAR subunit components.

In western blot analysis, we also found the up regulation of the phosphorylated NR2A (Tyr 1325) except the down regulation of the phosphorylated NR2B (Tyr1472) in GLYX-13 treatment group. While in patch clamp experiments, the significant suppression of the NR2B-containing NMDAR-mediated EPSC was observed but not obvious alteration in that of NR2A after GLYX-13 treatment. The discrepancy is mainly attributable to the difference between the *in vivo* MCAO model and OGD-induced *in vitro* model, including different experimental protocols of the intervention of GLYX-13, experimental mice of different age weeks and different mechanisms underlying GLYX-13’s effect at different post-ischemia stage.

In FJC staining, the number of FJC positive neurons in GLYX-13 treated group in DG was significant less than CA3 and CA1. We are not sure why it produced such an interesting result. Especially it occurred in DG. In the adult CNS, neurogenesis was limited in the subventricular zone of the lateral ventricles and the subgranular zone of the hippocampal DG. These neurogenic regions harbor a lot of neural progenitor cells, which can continue to proliferate and differentiate. One study (Namba et al., 2009) showed that memantine (an uncompetitive NMDAR antagonist) increases the cell proliferation and promotes the maturation of neuron. Whether or not NMDAR modulator GLYX-13 also plays a role in neurogenesis of DG, which is unknown. We will do further research on this phenomenon in the future.

GLYX-13 is currently used as an anti-depressant drug. More importantly, no GLYX-13 treatment related severe adverse side events occurred during the clinical study (Moskal et al., 2014). No difference (including vital signs, ECG, oxygen saturation and laboratory or hematological data) was observed between treatment group and control group (Moskal et al., 2014). There are approximately 5% subjects which reported mild or moderate side effects (Moskal et al., 2014). These included dizziness, headache, somnolence, fatigue and dysgeusia. Unlike other NMDAR modulators studied in human beings, GLYX-13 did not increase BPRS+ score (psychotomimetic effects) following administration at any time (Moskal et al., 2014). Therefore, GLYX-13 appears promising in clinical application.

In conclusion, the present study investigated the neuroprotective effects of glycine-site partial agonist of NMDAR GLYX-13 *in vivo* MCAO model and *in vitro* OGD-induced model. Our study shows that GLYX-13 attenuated i-LTP after ischemia by differential modulations of NMDAR subunit components at different post-ischemia stage. As a possible mechanism for preventing i-LTP, thereby reducing the NR2B-containing NMDAR-mediated excitotoxicity. We will further investigate the long-term efficacy of GLYX-13 for stroke treatment in a future study. In addition, GLYX-13 is currently used as an anti-depressant drug and its safety is proven. Thus our study suggests that GLYX-13 should be a promising neuroprotective agent for stroke patients.

## Author Contributions

HS: acquisition of funding; HS and CZ designed this study; CZ wrote the manuscript; CZ, ZHQ, MZH, NNL and BF carried out the experiments; RD, YYL and LPW analyzed the data; ALL did some experimental preparation.

## Conflict of Interest Statement

The authors declare that the research was conducted in the absence of any commercial or financial relationships that could be construed as a potential conflict of interest.

## Abbreviations

DG: dentate gyrus
NMDARs: NMDA receptors
i-LTP: ischemic long-term potentiation
MCAO: middle cerebral artery occlusion
OGD: oxygen-glucose deprivation
TTC: triphenyltetrazolium chloride.
